# Positive Emotion Dysregulation: A Metacognitive Perspective

**DOI:** 10.1002/cpp.70109

**Published:** 2025-06-26

**Authors:** Giovanni Mansueto, Sara Palmieri, Lucia Salatini, Sofia Piccioni, Giovanni Maria Ruggiero, Sandra Sassaroli, Marcantonio M. Spada, Gabriele Caselli

**Affiliations:** ^1^ Department of Health Sciences University of Florence Florence Italy; ^2^ School of Applied Sciences London South Bank University London UK; ^3^ Department of Psychology Sigmund Freud University Milan Italy; ^4^ Cognitive Psychotherapy School and Research Center Studi Cognitivi Milan Italy

**Keywords:** emotion dysregulation, emotion regulation, metacognitions, metacognitive, positive emotion dysregulation

## Abstract

**Introduction:**

Using the metacognitive model of emotion dysregulation as a basis, this study explored whether metacognitive beliefs are associated with positive emotion dysregulation.

**Method:**

A total of 436 participants from the general population and 133 outpatients seeking psychological treatment were recruited. Positive emotion dysregulation, metacognitive beliefs, and affective symptoms were assessed. T*t* test, chi‐square test, correlation, and hierarchical multiple linear regression analyses were run.

**Results:**

In the general population, over and above age, sex, and affective symptoms, a higher endorsement on positive beliefs about worry, negative beliefs about thoughts concerning uncontrollability and danger, and beliefs about the need to control thoughts were associated with a poor acceptance of positive emotions (*F* = 13.66, *p* < 0.001), with difficulties in engaging in goal‐directed behavior (*F* = 9.06, *p* < 0.001), and with difficulties in controlling behaviors when experiencing positive emotions (*F* = 10.61, *p* < 0.001). In the clinical sample, over and above age, sex, and affective symptoms, a higher endorsement of negative beliefs about thoughts concerning uncontrollability and danger and lack of cognitive confidence were associated with difficulties in engaging in goal‐directed behavior (*F* = 5.74, *p* < 0.001) and with difficulties in controlling behaviors when experiencing positive emotions (*F* = 6.54, *p* < 0.001). Outpatients seeking psychological treatment also reported more severe positive emotion dysregulation and higher endorsement of metacognitive beliefs when compared with the general population (*p* < 0.001).

**Conclusion:**

Positive emotion dysregulation appears to be associated with the tendency to endorse metacognitive beliefs. Metacognitive beliefs could be a potential therapeutic target for reducing difficulties in the regulation of positive emotions.

**Summary:**

- Among participants from the general population, the tendency to endorse metacognitive beliefs is associated with greater positive emotion dysregulation.
- Among outpatients seeking psychological treatment, the tendency to endorse metacognitive beliefs is associated with greater positive emotion dysregulation.
- Assessing metacognitive beliefs may allow clinicians to gain a clearer understanding of treatment trajectories to reduce difficulties in the regulation of positive emotions.
- Metacognitive beliefs could be the potential therapeutic target to reduce positive emotion dysregulation.
- Metacognitive Therapy techniques could be suitable approaches to reduce positive emotion dysregulation.

## 1 Introduction

During the two last decades, there has been a significant growth in research on emotion dysregulation. Emotion dysregulation refers to difficulties in controlling one's own behavior in the context of emotions (Gratz and Roemer 2004; Weiss, Darosh, et al. 2019). It is a multifaceted construct involving maladaptive ways of responding to emotions, including (a) lack of emotional awareness, clarity, and acceptance; (b) behavioral dyscontrol in the context of intense emotions; (c) unwillingness to pursue meaningful activities in the context of emotional distress; and (d) inflexible use of adaptive strategies to modulate (vs. eliminate) the intensity and/or duration of emotional experiences (Gratz and Roemer 2004). Studies have primarily focused on emotion dysregulation stemming from negative emotions (Gratz and Roemer 2004; Weiss, Darosh, et al. 2019), highlighting the dysregulation of negative emotions as a transdiagnostic vulnerability factor in psychological distress, health risky behaviors, and psychological disorders (Aldao et al. 2010; Brockmeyer et al. 2014; Gratz and Roemer 2004; Sloan et al. 2017; Weiss, Darosh, et al. 2019; Weiss et al. 2022).

Similarly to the difficulties in regulating negative emotions, evidence has suggested that individuals may also struggle with regulating positive emotions (Du Pont et al. 2016; Weiss et al. 2015; Weiss, Darosh, et al. 2019). In 2015, Weiss and colleagues developed a validated measure of clinically relevant difficulties related to the regulation of positive emotions, that is, the Difficulties in Emotion Regulation Scale‐Positive (DERS‐P). Positive emotion dysregulation was defined as a multifaceted construct involving maladaptive ways of responding to one's own positive emotions, including (a) non‐acceptance of positive emotions, characterized by the tendency to judge positive emotion states as undesirable, unpredictable, and/or frightening; (b) difficulties engaging in goal‐directed behavior when experiencing positive emotions; and (c) difficulties controlling behaviors when experiencing positive emotions, characterized by the tendency to engage in rash actions in the context of positive emotional experiences (Weiss et al. 2015; Weiss, Darosh, et al. 2019). Positive emotion dysregulation has been reported to be associated with a wide range of negative clinical outcomes, including impulsive behavior (Rogier et al. 2022; Waite et al. 2024; Weiss, Darosh, et al. 2019), emotional avoidance (Weiss, Darosh, et al. 2019), more severe PTSD symptoms (Weiss, Darosh, et al. 2019; Weiss et al. 2020), eating psychopathology (Howells et al. 2024; Santos and Haynos 2023), addictive behaviors and substance misuse (Rogier et al. 2022; Weiss et al. 2018, 2022; Weiss, Darosh, et al. 2019), and affective symptoms (Raudales et al. 2021; Santos and Haynos 2023; Weiss, Darosh, et al. 2019; Weiss, Nelson, et al. 2019).

Based on this evidence, it has been purported that identifying maintenance mechanisms of positive emotion dysregulation is important as it may allow for the identification of modifiable factors that could be targeted in clinical interventions (Weiss et al. 2015; Weiss, Darosh, et al. 2019). However, a comprehensive understanding of the underlying mechanisms in positive emotion dysregulation is still lacking (Weiss et al. 2015; Weiss, Darosh, et al. 2019). Among cognitive and behavioral models, the metacognitive model of emotion dysregulation (Mansueto, Jarach, et al. 2024), grounded in the self‐regulation executive function (S‐REF) theory (Wells and Matthews 1996), may be able to offer insights into potential maintenance factors of positive emotion dysregulation (Mansueto, Jarach, et al. 2024). The metacognitive model of emotion dysregulation proposes that difficulties in emotion regulation are linked to maladaptive metacognitive beliefs (Mansueto, Jarach, et al. 2024), that is, beliefs that individuals hold about their own internal states (i.e., thoughts and emotions) and about coping strategies that impact these states (Wells and Matthews 1996). Consistent with the metacognitive perspective, the tendency to endorse unhelpful metacognitive beliefs may lead to emotion dysregulation (Mansueto, Jarach, et al. 2024; Wells and Matthews 1996).

Research on the relationship between metacognitive beliefs and emotion dysregulation has, to date, focused exclusively on emotion dysregulation stemming from negative emotions (Mansueto, Jarach, et al. 2024). Literature has shown that a higher endorsement of metacognitive beliefs is associated with greater difficulties in the regulation of negative emotions across an array of populations, including general populations (Mansueto, Jarach, et al. 2024), outpatients with emotional disorders and comorbid personality disorders (Mansueto et al. 2022), outpatients with eating disorders (Palmieri et al. 2023), substance users (Mansueto, Palmieri, Sassaroli, et al. 2024), and those presenting with addictive behaviors (Mansueto, Jarach, et al. 2024).

Based on this evidence, which suggests that metacognitive beliefs could serve as maintenance factors in the dysregulation of negative emotions (Mansueto, Jarach, et al. 2024), it could be expected that a higher endorsement on metacognitive beliefs may be associated also with difficulties in the regulation of positive emotions (Mansueto, Jarach, et al. 2024). To date, no studies have investigated the association between metacognitive beliefs and positive emotion dysregulation. Understanding these relationships could provide insight into whether metacognitive beliefs are suitable therapeutic targets toward the reduction of difficulties in the regulation of positive emotions.

### 1.1 Aims

Within the framework of the metacognitive model of emotion dysregulation (Mansueto, Jarach, et al. 2024), the current study aimed to extend our understanding of underlying mechanisms of positive emotion dysregulation (Weiss et al. 2015; Weiss, Darosh, et al. 2019) by exploring whether metacognitive beliefs could act as potential maintenance factors of difficulties in the regulation of positive emotions. We aimed to explore the association between metacognitive beliefs and positive emotion dysregulation in a general population (Study 1) and in a clinical population (i.e., outpatients seeking psychological treatment) (Study 2). Using two different samples may help to observe the interplay among the study variables to further elucidate the role of metacognitive beliefs as possible maintenance factors of positive emotion dysregulation in both non‐clinical and clinical samples (Mansueto et al. 2022). We hypothesized that (a) among the general population a higher endorsement on metacognitive beliefs would be positively associated with positive emotion dysregulation; (b) among outpatients seeking psychological treatment a higher endorsement of metacognitive beliefs would be positively associated with positive emotion dysregulation; (c) as a corollary that the sample of outpatients seeking psychological treatment would endorse more strongly metacognitive beliefs and report higher levels of positive emotion dysregulation compared with the general population sample.

## 2 Study 1: Exploring the Relationship Between Metacognitive Beliefs and Emotion Dysregulation in Participants From General Population

### 2.1 Method

#### 2.1.1 Participants

A convenience sample of participants was recruited from the Italian general population, via email and social media. Participants were eligible for inclusion in the study if they were (a) 18 years of age or above, (b) able to provide informed consent, and (c) able to complete the online batch of self‐report measures in Italian. A total of 436 individuals were eligible. The exclusion criterion was the evidence of cognitive deficits or problems affecting the ability of reading, understanding, and following the study assessment process. Ethics approval for the study was obtained from the ethics committee of Sigmund Freud University, Milan, Italy (no. NCKTL89MBJSK7889926). All procedures contributing to this work comply with the ethical standards of the relevant national and institutional committees on human experimentation and with the Helsinki Declaration of 1975, as revised in 2008. All participants provided a signed informed consent.

#### 2.1.2 Measures

Socio‐demographic and clinical information were collected via a set of interview‐based screening questions already used in the past (Mansueto et al. 2016, 2022).

Positive emotion dysregulation was measured with the Difficulties in Emotion Regulation Scale—Positive (DERS‐P) (Weiss et al. 2015, 2019a), a 13‐item self‐report measure. The DERS‐P is composed by three sub‐scales: non‐acceptance of positive emotions (DERS‐P Accept) (e.g., “When I'm happy, I become scared and fearful of those feelings”), difficulties in engaging in goal‐directed behaviors in the context of positive emotions (DERS‐P Goals) (e.g., “When I'm happy, I have difficulty focusing on other things”), and difficulties in controlling impulsive behaviors when experiencing positive emotions (DERS‐P Impulse) (e.g., “When I'm happy, I have difficulty controlling my behaviors”). The items are rated on a 5‐point Likert scale (from 1 = *almost never* to 5 = *almost always*). Higher scores show greater difficulties in regulating positive emotions (Weiss et al. 2015). The DERS‐P has been shown to possess good psychometric properties (Velotti et al. 2020; Weiss, Darosh, et al. 2019).

Metacognitive beliefs were measured with the Meta‐Cognitions Questionnaire 30 (MCQ‐30) (Wells and Cartwright‐Hatton 2004), a 30 item self‐report measure assessing individual differences in metacognitive beliefs, judgments, and monitoring tendencies. The MCQ‐30 is composed by five subscales: positive beliefs about worry (MCQ‐30 POS) (e.g., “Worry/rumination helps me cope”), negative beliefs about thoughts concerning uncontrollability and danger (MCQ‐30 NEG) (e.g., “When I start worrying I cannot stop”; “If I continue to ruminate I will lose my mind”), cognitive confidence (MCQ‐30 CC) (e.g., “My memory can mislead me at times”), beliefs about the need to control thoughts (MCQ‐30 NC) (e.g., “Not being able to control my thoughts is a sign of weakness”), and cognitive self‐consciousness (MCQ‐30 CSC) (e.g., “I pay close attention to the way my mind works”). The items are rated on a 4‐point Likert scale (from 1 = *I do not agree* to 4 = *I totally agree*). Higher scores indicate higher levels of maladaptive metacognitive beliefs. The MCQ‐30 has shown good psychometric properties (Quattropani et al. 2014; Wells and Cartwright‐Hatton 2004).

Affective symptoms were evaluated with the Generalized Anxiety Disorder 7 (GAD‐7; Spitzer et al. 2006) and the Patient Health Questionnaire 9 (PHQ‐9; Kroenke et al. 2001). The GAD‐7 (Spitzer et al. 2006) is a 7‐item self‐report measure assessing and screening generalized anxiety disorder and its severity during the past 2 weeks. The items are rated on a 4‐point Likert scale (from 0 = *not at all* to 3 = *nearly every day*). High scores indicate higher levels of anxiety. Scores ranging from 10 to 14 indicate generalized anxiety of moderate severity, and scores ranging from 15 to 21 indicate severe generalized anxiety. The GAD‐7 has been shown to possess good psychometric properties (Spitzer et al. 2006). The PHQ‐9 (Kroenke et al. 2001) is a 9‐item self‐ report measure assessing, screening, and monitoring depression severity during the past 2 weeks. The items are rated on a 4‐point Likert scale (from 0 = *not at all* to 3 = *nearly every day*). High scores indicate higher levels of depression. Scores ranging from 15 to 19 indicate depression of moderate severity, and scores ranging from 20 to 27 indicate severe depression. The PHQ‐9 has been shown to possess good psychometric and proprieties (Kroenke et al. 2001; Manea et al. 2015; Mazzotti et al. 2003).

#### 2.1.3 Statistical Analyses

First, descriptive analyses and univariate and multivariate normality tests were calculated. Skewness and kurtosis were considered acceptable for a linear model of analysis in a range of ± 3 (Mayers 2013). Second, correlation analyses were conducted to explore the associations among metacognitive beliefs, affective symptoms, and positive emotion dysregulation. Third, hierarchical linear regression models were run to evaluate whether metacognitive beliefs would predict positive emotion dysregulation. Only those variables showing a significant correlation with positive emotion dysregulation were included in the regression models as independent variables. Four adjustment variables were selected based on the literature, that is, sex, age, anxiety, and depressive symptoms (Mansueto et al. 2022; Mansueto, Palmieri, Sassaroli, et al. 2024; Weiss, Nelson, et al. 2019). The ordering of independent variables in the hierarchical multiple linear regression analyses was defined according to the causal structure suggested by the metacognitive model of emotion dysregulation (Mansueto, Jarach, et al. 2024; Wells and Matthews 1996): Sex and age were entered in the first step, anxiety and depressive symptoms were entered in the second step, metacognitive beliefs were entered in the third step. The dependent variable was emotion dysregulation (i.e., DERS‐P total score and DERS‐P subscales scores; Weiss, Nelson, et al. 2019). Statistical assumptions for using hierarchical linear regression analyses were evaluated (Barbaranelli and D'Olimpio 2006; Field 2013; Myers 1990). Multicollinearity statistics were within acceptable ranges (Tolerance Index ranged from 0.40 to 0.99; Variance Inflation Factor ranged from 1.001 to 2.86) (Barbaranelli and D'Olimpio 2006; Bowerman and O'Connell 1990; Field 2013; Hair et al. 1998). The Durbin–Watson test (ranged from 2.02 to 2.09) showed that there were no significant correlations between standardized residuals and independent variables (Barbaranelli and D'Olimpio 2006; Field 2013). Effect size was calculated via Cohen's *f* ^2^ (Cohen 1998; Coolican 2009; Ialongo 2016). The two‐sided significance level was set at *p* ≤ 0.05. Statistical analyses were run using SPSS version 29 of SPSS (IBM SPSS Statistics).

### 2.2 Results

#### 2.2.1 Descriptive Statistics for Participants From the General Population

Table 1 shows socio‐demographic characteristics of the sample. A total of 436 participants were enrolled. The mean age was 18.01 years (SD = 5.53); 320 (73.4%) participants were females, and 116 (26.6%) were males. Regarding education, 11 participants (2.5%) had a secondary school diploma, 117 (26.9%) had a high‐school diploma, 119 (27.3%) had bachelor's degrees, 162 (37.2%) had master's degrees, and the remaining 27 participants (6.2%) had post–master's degrees. The majority of participants (*n* = 357, 81.9%) were unmarried, while the remaining were married (*n* = 79, 18.1%). Regarding working status, 16 (3.7%) participants were unemployed, 292 (67.9%) were employed, 120 (27.5%) comprised university students, and the remaining were retired (*n* = 4, 0.9%). Table 2 shows mean and standard deviations for positive emotion dysregulation, metacognitive beliefs, and the severity of affective symptoms.

**TABLE 1:** Comparisons between participants from general population and outpatients with affective disturbance and personality disorders on socio‐demographic variables.

	General population (*n* = 436)	Outpatients with affective disturbance and personality disorders (*n* = 133)			
	M (SD)	M (SD)	*t*_(df)_	*p*	Cohen's *d*
Age	17.59 (5.53)	19.82 (7.31)	−0.18_(567)_	0.427	0.018
	***n* (%)**	***n* (%)**	***χ*** ^**2**^_**(df)**_	***p***	**Cramer's *V***
Sex					
Female	320 (73.4%)	83 (62.4%)	5.95_(1)_	0.015	0.102
Male	116 (26.6%)	50 (37.6%)			
Education					
Primary school diploma	0 (0.00%)	0 (0.00%)	—	—	—
Secondary school diploma	11 (2.5)	4 (3.1%)	15.09_(4)_	0.005	0.163
High school diploma	117 (26.9%)	52 (39.7%)			
Bachelor's degree	119 (27.3%)	39 (29.8%)			
Master's degree	162 (37.2%)	35 (26.7%)			
Post–master's degree	27 (6.2%)	1 (0.8%)			
Civil Status					
Unmarried	357 (81.9%)	111 (83.5%)	0.67_(1)_	0.795_a_	0.017
Married	79 (18.1%)	22 (16.5%)			
Working status					
Unemployed	16 (3.7%)	13 (9.8%)	9.07_(3)_	0.028	0.126
Employed	292 (67.9%)	89 (23.1%)			
Student	120 (27.5%)	29 (21.8%)			
Retired	4 (0.9%)	2 (1.5%)			

*Note:* a = Fisher's test.

**TABLE 2:** Comparisons between participants from the general population and outpatients with affective disturbance and personality disorders on positive emotion dysregulation, metacognitive beliefs, and affective symptoms.

	General population (*n* = 436)	Outpatients with affective disturbance and personality disorders (*n* = 133)	*t*_(df)_	*p*	Cohen's *d*
	M (SD)	M (SD)			
DERS‐P	17.59 (5.53)	19.82 (7.31)	−3.75_(567)_	< 0.001	0.37
DERS‐P‐A	4.81 (1.72)	5.26 (2.25)	−2.44_(567)_	0.007	0.24
DERS‐P‐G	6.44 (2.66)	7.12 (2.96)	−2.49_(567)_	0.007	0.25
DERS‐P‐I	6.33 (2.34)	7.44 (3.53)	−4.17_(567)_	< 0.001	0.41
MCQ‐30 POS	10.68 (3.91)	11.35 (4.38)	−1.67_(567)_	0.047	0.17
MCQ‐30 NEG	13.23 (4.21)	15.08 (3.82)	−6.27_(567)_	< 0.001	0.62
MCQ‐30 CC	11.53 (4.49)	12.15 (5.12)	−1.32_(567)_	0.092	0.13
MCQ‐30 NC	10.87 (3.49)	12.28 (4.24)	−3.86_(567)_	< 0.001	0.38
MCQ‐30 CSC	15.24 (3.56)	15.57 (3.75)	−0.94_(567)_	0.173	0.09
GAD‐7	7.48 (4.75)	10.45 (4.76)	−8.67_(567)_	< 0.001	0.85
PHQ‐9	7.12 (4.96)	11.64 (6.09)	−6.28_(567)_	< 0.001	0.66

Abbreviations: DERS‐P: Difficulties in Emotion Regulation Scale‐Positive—dysregulation of positive emotion; DERS‐P‐A: Difficulties in Emotion Regulation Scale‐Positive—Acceptance; DERS‐P‐G: Difficulties in Emotion Regulation Scale‐Positive—Goals; DERS‐P‐I: Difficulties in Emotion Regulation Scale‐Positive—Impulse; GAD‐7: General Anxiety Disorder‐7; MCQ‐30—CC: Metacognitions Questionnaire‐30—Cognitive Confidence; MCQ‐30—CSC: Metacognitions Questionnaire‐30—Cognitive Self‐Consciousness; MCQ‐30—NC: Metacognitions Questionnaire‐30—Beliefs about the Need to Control Thoughts; MCQ‐30—NEG: Metacognitions Questionnaire‐30—Negative Beliefs about Thoughts concerning Uncontrollability and Danger; MCQ‐30—POS: Metacognitions Questionnaire‐30—Positive Beliefs about Worry; PHQ‐9: Patient Health Questionnaire‐9.

#### 2.2.2 Metacognitive Beliefs and Dysregulation of Positive Emotions Among the General Population Sample

Correlation analyses exploring the association between metacognitive beliefs, affective symptoms, and positive emotion dysregulation are reported in Table 3. A hierarchical multiple linear regression model exploring the role of metacognitive beliefs in predicting positive emotional dysregulation is reported in Table 4. The criterion variable in the hierarchical regression models was positive emotion dysregulation (DERS‐P total score). A closer inspection of the final equation indicates that, over and above age, sex, anxiety, and depressive symptoms, positive beliefs about worry and negative beliefs about thoughts concerning uncontrollability and danger were significantly and positively associated with positive emotion dysregulation (*F* = 15.56, df = 9, *p* < 0.001, Cohen's *f* ^2^ = 0.33). Hierarchical multiple linear regression models exploring the association between metacognitive beliefs and subcomponents of positive emotional dysregulation (DERS‐P subscales scores) are reported in Table 5. Hierarchical regression models showed that, adjusting for age, sex, anxiety, and depressive symptoms: (a) positive beliefs about worry, negative beliefs about thoughts concerning uncontrollability, and beliefs about the need to control thoughts were significantly associated with higher levels of non‐acceptance of positive emotions (DERS‐P‐A) (*F* = 13.66, df = 9, *p* < 0.001, *r* ^2^ = 0.224, Cohen's *f* ^2^ = 0.29); (b) positive beliefs about worry and negative beliefs about thoughts concerning uncontrollability were significantly associated with greater difficulties in engaging in goal‐directed behavior when experiencing positive emotions (DERS‐P‐G) (*F* = 9.06, df = 9, *p* < 0.001, *r* ^2^ = 0.161, Cohen's *f* ^2^ = 0.19); and (c) positive beliefs about worry and negative beliefs about thoughts concerning uncontrollability were significantly associated with greater difficulties in controlling behaviors when experiencing positive emotions (DERS‐P‐I) (*F* = 10.61, df = 9, *p* < 0.001, *r* ^2^ = 0.183, Cohen's *f* ^2^ = 0.22).

**TABLE 3:** Intercorrelations among study variables among participants from the general population (*n* = 436).

		1	2	3	4	5	6	7	8	9	10	11
1	DERS‐P	1										
2	DERS‐P‐A	0.72***	1									
3	DERS‐P‐G	0.83***	0.35***	1								
4	DERS‐P‐I	0.88***	0.58***	0.57***	1							
5	MCQ‐30 POS	0.25***	0.22***	0.18***	0.24***	1						
6	MCQ‐30 NEG	0.42***	0.40***	0.32***	0.35***	0.16***	1					
7	MCQ‐30 CC	0.26***	0.15***	0.23***	0.24***	0.21***	0.42***	1				
8	MCQ‐30 NC	0.35***	0.38***	0.20***	0.34***	0.35***	0.65***	0.36***	1			
9	MCQ‐30 CSC	0.19***	0.16***	0.15***	0.18***	0.34***	0.44***	0.07	0.43***	1		
10	PHQ‐9	0.39***	0.32***	0.31***	0.33***	0.26***	0.58***	0.42***	0.48***	0.28***	1	
11	GAD‐7	0.38***	0.39***	0.29***	0.31***	0.25***	0.67***	0.36***	0.51***	0.30***	0.76***	1

Abbreviations: DERS‐P: Difficulties in Emotion Regulation Scale‐Positive—dysregulation of positive emotion; DERS‐P‐A: Difficulties in Emotion Regulation Scale‐Positive—Acceptance; DERS‐P‐G: Difficulties in Emotion Regulation Scale‐Positive—Goals; DERS‐P‐I: Difficulties in Emotion Regulation Scale‐Positive—Impulse; GAD‐7: General Anxiety Disorder‐7; MCQ‐30—CC: Metacognitions Questionnaire‐30—Cognitive Confidence; MCQ‐30—CSC: Metacognitions Questionnaire‐30—Cognitive Self‐Consciousness; MCQ‐30—NC: Metacognitions Questionnaire‐30—Beliefs about the Need to Control Thoughts; MCQ‐30—NEG: Metacognitions Questionnaire‐30—Negative Beliefs about Thoughts concerning Uncontrollability and Danger; MCQ‐30—POS: Metacognitions Questionnaire‐30—Positive Beliefs about Worry; PHQ‐9: Patient Health Questionnaire‐9.

*** *p* < 0.001. ** *p* < 0.01, * *p* < 0.05.

**TABLE 4:** Hierarchical regression model examining the role of metacognitive beliefs in predicting positive emotion dysregulation among participants from the general population (*n* = 436).

	B (SE)	*β*	*p*	95% CI	*R* ^2^	Adj *R* ^2^	Δ*R* ^2^
**Step 1**					0.024	0.019	
Age	−0.07 (0.02)	−0.15	0.001	−0.12 to 0.03			
Sex	0.38 (0.59)	0.03	0.522	−0.78 to 1.54			
**Step 2**					0.182	0.175	0.158
Age	−0.04 (0.02)	−0.08	0.049	−0.08 to 0.00			
Sex	0.71 (0.54)	0.05	0.19	−0.36 to 1.79			
PHQ‐9	0.21 (0.07)	0.18	0.006	0.05 to 3.53			
GAD‐7	0.29 (0.07)	0.25	< 0.001	0.13 to 0.44			
**Step 3**					0.247	0.232	0.065
Age	−0.03(0.02)	−0.07	0.097	−0.07 to 0.01			
Sex	0.56 (0.55)	0.04	0.310	−0.52 to 1.64			
PHQ‐9	0.11 (0.07)	0.11	0.117	−0.03 to 0.26			
GAD‐7	0.07 (0.08)	0.08	0.348	−0.08 to 0.24			
MCQ‐POS	0.22 (0.09)	0.15	0.002	0.08 to0.35			
MCQ‐NEG	0.36 (0.09)	0.27	< 0.001	0.17 to 0.54			
MCQ‐CC	0.04 (0.06)	0.03	0.517	−0.08 to 0.16			
MCQ‐NC	0.07 (0.09)	0.05	0.431	−0.11 to 0.26			
MCQ‐CSC	−0.09 (0.08)	−0.06	0.222	−0.22 to 0.06			

Abbreviations: GAD‐7: General Anxiety Disorder‐7; MCQ‐30–CC = Metacognitions Questionnaire‐30–Cognitive Confidence; MCQ‐30–CSC = Metacognitions Questionnaire‐30–Cognitive Self‐Consciousness; MCQ‐30–NC = Metacognitions Questionnaire‐30–Beliefs about the Need to Control Thoughts; MCQ‐30–NEG = Metacognitions Questionnaire‐30–Negative Beliefs about Thoughts concerning Uncontrollability and Danger; MCQ‐30–POS = Metacognitions Questionnaire‐30–Positive Beliefs about Worry; PHQ‐9: Patient Health Questionnaire‐9.

**TABLE 5:** Hierarchical regression models examining the role of metacognitive beliefs in predicting sub‐components of positive emotion dysregulation among participants from general population (*n* = 436).

	DERS‐P‐A		DERS‐P‐G		DERS‐P‐I	
	B (SE)	95% CI	B (SE)	95% CI	B (SE)	95% CI
**Step 1**						
Age	−0.01 (0.01)	−0.03 to 0.001	−0.04 (0.01)	−0.06 to 0.02	to 0.02 (0.01)	−0.04 to 0.001
Sex	−0.10 (0.18)	−0.47 to 0.26	0.17 (0.28)	−0.38 to 0.73	0.32 (0.25)	−0.18 to 0.82
**Step 2**						
Age	−0.01 (0.01)	−0.02 to 01	−0.03 (0.01)	−0.05 to 0.09	−0.01 (0.01)	−0.02 to 0.01
Sex	0.02 (0.17)	−0.32 to 0.36	0.27 (0.27)	−0.26 to 0.81	0.42 (0.24)	−0.05 to 0.89
PHQ‐9	0.02 (0.02)	0.07 to 0.17	0.09 (0.03)	0.02 to 0.16	0.09 (0.03)	0.03 to 0.16
GAD‐7	0.13 (0.02)	0.07 to 0.17	0.08 (0.03)	0.003 to 0.15	0.08 (0.03)	0.01 to 0.15
**Step 3**						
Age	−0.002 (0.01)	−0.01 to 0.01	−0.03 (0.01)	−0.05 to −0.01	−0.01 (0.01)	−0.02 to 0.01
Sex	−0.03 (0.17)	−0.37 to 0.30	0.29 (0.28)	−0.25 to 0.84	−0.30 (0.24)	−0.17 to 0.78
PHQ‐9	0.001 (0.02)	−0.04 to 0.05	0.06 (0.04)	−0.01 to 0.13	0.05 (0.03)	−0.01 to 0.12
GAD‐7	0.06 (0.03)	0.01 to 0.11	0.01 (0.04)	−0.07 to 0.10	0.001 (0.03)	−0.07 to 0.07
MCQ‐30 POS	0.06 (0.02)	0.10 to 0.76	0.08 (0.03)	0.009 to 0.14	0.08 (0.03)	0.02 to 0.14
MCQ‐30 NEG	0.08 (0.03)	0.15 to 0.35	0.16 (0.05)	0.06 to 0.25	0.11 (0.04)	0.19 to 0.34
MCQ‐30 CC	−0.03 (0.02)	−0.07 to 0.004	0.05 (0.03)	−0.01 to 0.12	0.02 (0.03)	−0.03 to 0.07
MCQ‐30 NC	0.09 (0.03)	0.03 to 0.15	−0.08 (0.05)	−0.18 to 0.01	0.06 (0.04)	−0.01 to 0.15
MCQ‐30 CSC	−0.04 (0.02)	−0.09 to 0.002	−0.02 (0.04)	−0.09 to 0.06	−0.03 (0.03)	−0.10 to 0.04

Abbreviations: DERS‐P‐A: Difficulties in Emotion Regulation Scale‐Positive—Acceptance; DERS‐P‐G: Difficulties in Emotion Regulation Scale‐Positive—Goals; DERS‐P‐I: Difficulties in Emotion Regulation Scale‐Positive—Impulse; GAD‐7: General Anxiety Disorder‐7; MCQ‐30—CC = Metacognitions Questionnaire‐30—Cognitive Confidence; MCQ‐30—CSC = Metacognitions Questionnaire‐30—Cognitive Self‐Consciousness; MCQ‐30—NC = Metacognitions Questionnaire‐30—Beliefs about the Need to Control Thoughts; MCQ‐30—NEG = Metacognitions Questionnaire‐30—Negative Beliefs about Thoughts concerning Uncontrollability and Danger; MCQ‐30—POS = Metacognitions Questionnaire‐30—Positive Beliefs about Worry; NC: no significant correlations among variables; PHQ‐9: Patient Health Questionnaire‐9.

## 3 Study 2: Exploring the Relationship Between Metacognitive Beliefs and Emotion Dysregulation in Participants From the Clinical Sample

### 3.1 Method

#### 3.1.1 Participants

Participants were outpatients seeking psychological treatment at the private clinical center Studi Cognitivi (Italy). Study inclusion criteria were as follows: (a) 18 years of age or above, (b) being able to provide informed consent, (c) being an outpatient seeking psychological treatment for affective distress, and (d) completion of the clinician‐administered diagnostic interview (Structured Clinical Interview for DSM‐IV Axis I Disorders, SCID‐II, First et al. 1994; Mini International Neuropsychiatric Interview, M.I.N.I., Sheehan et al. 1998; SCID‐5 Personality Disorders, SCID‐5‐PD, First, Spitzer, et al. 2015; SCID‐5 Clinical Version, SCID‐5 CV, First, Williams, et al. 2015). A total of 133 participants were eligible. The procedure was described in detail elsewhere (Spada et al. 2021; Mansueto et al. 2022). The exclusion criterion was the evidence of cognitive deficits or problems affecting the ability to read, understand, and follow the study assessment process. Ethics approval for the study was obtained from the ethics committee of Sigmund Freud University, Milan, Italy (no. NCKTL89MBJSK7889926). All procedures contributing to this work comply with the ethical standards of the relevant national and institutional committees on human experimentation and with the Helsinki Declaration of 1975, as revised in 2008. All participants provided a signed informed consent.

#### 3.1.2 Measures

After obtaining informed consent, participants provided demographic details and completed the clinical administered diagnostic interview (Structured Clinical Interview for DSM‐IV Axis I disorders, SCID‐II, First et al. 1994; Mini International Neuropsychiatric Inter‐ view, M.I.N.I., Sheehan et al. 1998; SCID‐5 Personality Disorders, SCID‐ 5‐PD, First, Spitzer, et al. 2015; SCID‐5 Clinical Version, SCID‐5 CV, First, Williams, et al. 2015). They then completed the batch of self‐report measures. Positive emotion dysregulation, metacognitive beliefs, anxiety, and depressive symptoms were measured with the same measures described for the Study 1 in the general population sample, that is, DERS‐P (Weiss, Nelson, et al. 2019), MCQ‐30 (Wells and Cartwright‐Hatton 2004), GAD‐7 (Spitzer et al. 2006), and PHQ‐9 (Kroenke et al. 2001).

#### 3.1.3 Statistical Analyses

The two samples, that is, general population (see Study 1, Section 2.1.2) and clinical sample (Study 2), were compared with respect to demographic and clinical variables. Comparisons between the two samples were run via the chi‐square test or Fisher test, when > 20% of cells had expected frequencies < 5, in case of categorical variables (Cramer's *V* coefficient was calculated as a measure of effects size) (Cumming 2012), and via the *t* test for independent samples or via the Mann–Whitney test for normally or nonnormally distributed variables respectively (Cohen's *d* coefficient and *η* ^2^ were calculated as a measure of effects size for *t* test and Mann–Whitney test, respectively) (Coolican 2009; Cohen 1998; Fritz et al. 2012).

Then, the statistical procedure followed that described for Study 1 in the general population sample (see Study 1, Section 2.1.3). Correlation analyses were conducted in order to evaluate the associations among the study variables. Then hierarchical multiple linear regression models were run to evaluate whether metacognitive beliefs would predict positive emotion dysregulation. Only those variables showing a significant correlation with positive emotion dysregulation were included in the regression models as independent variables. The ordering of independent variables was as follows: Sex and age were entered in the first step, anxiety and depressive symptoms were entered in the second step, and metacognitive beliefs were entered in the third step (Mansueto, Jarach, et al. 2024; Wells and Matthews 1996). The dependent variable was emotion dysregulation (i.e., DERS‐P total score and DERS‐P subscales scores; Weiss, Nelson, et al. 2019). Statistical assumptions for using hierarchical linear regression analyses were evaluated (Barbaranelli and D'Olimpio 2006; Field 2013; Myers 1990). Multicollinearity statistics were within acceptable ranges (Tolerance Index ranged from 0.47 to 0.96; Variance Inflation Factor ranged from 1.03 to 2.24) (Barbaranelli and D'Olimpio 2006; Bowerman and O'Connell 1990; Field 2013; Hair et al. 1998). The Durbin–Watson test (ranged from 1.84 to 2.11) showed that there were no significant correlations between standardized residuals and independent variables (Barbaranelli and D'Olimpio 2006; Field 2013). Effect size was calculated via Cohen's *f* ^2^ (Cohen 1998; Coolican 2009; Ialongo 2016). The two‐sided significance level was set at *p* ≤ 0.05. Statistical analyses were run using SPSS version 29 of SPSS (IBM SPSS Statistics).

### 3.2 Results

#### 3.2.1 Descriptive Statistic Among Clinical Samples

Table 1 shows socio‐demographic characteristic of the sample. A total of 133 participants were enrolled. The mean age was 19.82 years (SD = 7.31), 83 (62.4%) were females, and 50 (37.6%) were males. Regarding education, four participants (3.1%) had secondary school diploma, 52 (39.7%) had had a high‐school diploma, 39 (29.8%) had bachelor's degrees, 35 (26.7%) had master's degrees, and three participants (0.2%) had a post–master's degree. The majority of participants (*n* = 111, 83.5%) were unmarried, while the remaining were married (*n* = 22, 16.5%). Regarding the working status, 13 (9.8%) participants were unemployed, 89 (23.1%) were employed, 29 (21.8%) comprised university students, and the remaining were retired (*n* = 2, 1.5%). Concerning the prevalence of psychological diseases, 46 (34.58%) participants had mood disorders, 51 had anxiety disorders (38.34%), 45 had personality disorders (33.83%), and 24 (18.05%) were outpatients with affective distress below the clinical threshold (see Table S1). Table 2 shows mean and standard deviation of levels of positive emotion dysregulation, metacognitive beliefs, and the severity of affective symptoms.

#### 3.2.2 Metacognitive Beliefs and Dysregulation of Positive Emotions in the Clinical Sample

Correlation analyses exploring the association between metacognitive beliefs, affective symptoms, and positive emotion dysregulation are reported in Table 6. A hierarchical multiple linear regression model exploring the role of metacognitive beliefs in predicting positve emotion dysregulation is reported in Table 7. A closer inspection of the final equation indicates that, over and above age, sex, anxiety, and depressive symptoms, negative beliefs about thoughts concerning uncontrollability and cognitive confidence were significantly and positively associated with positive emotion dysregulation (*F* = 9.21, df = 7, *p* < 0.001, Cohen's *f* ^2^ = 0.51).

**TABLE 6:** Intercorrelations among study variables in the outpatients with affective disturbance and personality disorders (*n* = 133).

		1	2	3	4	5	6	7	8	9	10	11
1	DERS‐P	1										
2	DERS‐P‐A	0.74***	1									
3	DERS‐P‐G	0.82***	0.39***	1								
4	DERS‐P‐I	0.90***	0.57***	061***	1							
5	MCQ‐30 POS	0.14	−0.03	0.18*	0.16	1						
6	MCQ‐30 NEG	0.44***	0.35***	0.37***	0.38***	0.17*	1					
7	MCQ‐30 CC	0.37***	0.16	0.39***	0.34***	0.19*	0.25**	1				
8	MCQ‐30 NC	0.41***	0.36***	0.31**	0.35***	0.32***	0.57***	0.31**	1			
9	MCQ‐30 CSC	0.02	0.01	0.04	0.01	0.15	0.31***	0.05	0.34***	1		
10	PHQ‐9	0.47***	0.39***	0.37***	0.41***	0.14	0.53***	0.41***	0.54***	0.16	1	
11	GAD‐7	0.27**	0.21*	0.23**	0.23**	0.26**	0.62***	0.13	0.48***	0.26**	0.59***	1

Abbreviations: DERS‐P: Difficulties in Emotion Regulation Scale‐Positive—dysregulation of positive emotion; DERS‐P‐A: Difficulties in Emotion Regulation Scale‐Positive—Acceptance; DERS‐P‐G: Difficulties in Emotion Regulation Scale‐Positive—Goals; DERS‐P‐I: Difficulties in Emotion Regulation Scale‐Positive—Impulse; GAD‐7: General Anxiety Disorder‐7; MCQ‐30—CC = Metacognitions Questionnaire‐30—Cognitive Confidence; MCQ‐30—CSC = Metacognitions Questionnaire‐30—Cognitive Self‐Consciousness; MCQ‐30—NC = Metacognitions Questionnaire‐30—Beliefs about the Need to Control Thoughts; MCQ‐30—NEG = Metacognitions Questionnaire‐30—Negative Beliefs about Thoughts concerning Uncontrollability and Danger; MCQ‐30—POS = Metacognitions Questionnaire‐30—Positive Beliefs about Worry; PHQ‐9: Patient Health Questionnaire‐9.

*** *p* < 0.001.

** *p* < 0.01.

* *p* < 0.05.

**TABLE 7:** Hierarchical regression model examining the role of metacognitive beliefs in predicting positive emotion dysregulation in outpatients with affective disturbance and personality disorders (*n* = 133).

	B (SE)	*β*	*p*	95% CI	*R* ^2^	Adj *R* ^2^	Δ*R* ^2^
**Step 1**					0.062	0.047	
Age	−0.16 (0.05)	−0.25	0.004	−0.26 to −0.05			
Sex	0.27 (1.29)	0.02	0.830	−2.28 to 2.84			
**Step 2**					0.237	0.213	0.175
Age	−0.08 (0.05)	−0.13	0.106	−0.18 to 0.18			
Sex	0.20 (1.19)	0.01	0865	−2.16 to 2.57			
PHQ‐9	0.53 (0.12)	0.44	< 0.001	0.30 to 0.76			
GAD‐7	−0.03 (0.15)	−0.01	0.848	−0.32 to 0.26			
**Step 3**					0.340	0.303	0.103
Age	−0.08 (0.05)	−0.13	0.092	−0.18 to 0.01			
Sex	0.17 (1.12)	0.01	0.878	−2.06 to 2.41			
PHQ‐9	0.28 (0.12)	0.24	0.025	0.03 to 0.54			
GAD‐7	−0.20 (0.16)	−0.13	0.200	−0.52 to 0.11			
MCQ‐30 NEG	0.52 (0.19)	0.27	0.010	0.12 to 0.90			
MCQ‐30 CC	0.29 (0.12)	0.20	0.017	0.05 to 0.52			
MCQ‐30 NC	0.16 (0.16)	0.09	0.346	−0.17 to 0.48			

Abbreviations: DERS‐P: Difficulties in Emotion Regulation Scale‐Positive—dysregulation of positive emotion; GAD‐7: General Anxiety Disorder‐7; MCQ‐30—CC = Metacognitions Questionnaire‐30—Cognitive Confidence; MCQ‐30—NC = Metacognitions Questionnaire‐30—Beliefs about the Need to Control Thoughts; MCQ‐30—NEG = Metacognitions Questionnaire‐30—Negative Beliefs about Thoughts concerning Uncontrollability and Danger; PHQ‐9: Patient Health Questionnaire‐9.

Hierarchical multiple linear regression models exploring the association between metacognitive beliefs and subcomponents of positive emotional dysregulation (DERS‐P sub‐scales scores) are reported in Table 8. Hierarchical regression models showed that, adjusting for age, sex, anxiety, and depressive symptoms: (a) metacognitive beliefs were not associated with difficulties in acceptance of positive emotions (DERS‐P‐A) (*F* = 5.06, df = 6, *p* < 0.001, *r* ^2^ = 0.221, Cohen's *f* ^2^ = 0.28); (b) negative beliefs about thoughts concerning uncontrollability and cognitive confidence were significantly associated with greater difficulties in engaging in goal‐directed behavior when experiencing positive emotions (DERS‐P‐G) (*F* = 5.74, df = 8, *p* < 0.001, *r* ^2^ = 0.270, Cohen's *f* ^2^ = 0.37); and (c) negative beliefs about thoughts concerning uncontrollability and cognitive confidence were significantly associated with greater difficulties in controlling behaviors when experiencing positive emotions (DERS‐P‐I) (*F* = 6.54, df = 7, *p* < 0.001, *r* ^2^ = 0.268, Cohen's *f* ^2^ = 0.37).

**TABLE 8:** Hierarchical regression models examining the role of metacognitive beliefs in predicting sub‐components of positive emotion dysregulation in outpatients with affective disturbance and personality disorders (*n* = 133).

	DERS‐P‐A		DERS‐P‐G		DERS‐P‐I	
	B (SE)	95% CI	B (SE)	95% CI	B (SE)	95% CI
**Step 1**						
Age	−0.03 (0.01)	−0.06 to 0.001	−0.05 (0.02)	−0.09 to −0.01	−0.07 (0.03)	−0.12 to 0.02
Sex	0.11 (0.41)	−0.69 to 0.91	0.07 (0.53)	−0.98 to 1.12	0.09 (0.63)	−1.15 to 1.34
**Step 2**						
Age	−0.01 (0.01)	−0.05 to 0.02	−0.03 (0.02)	−0.07 to 0.01	−0.04 (0.03)	−0 90 to 0.01
Sex	0.08 (038)	−0.68 to 0.85	0.07 (0.51)	−0.94 to 1.08	0.04 (0.59)	−1.13 to 1.23
PHQ‐9	0.14 (0.04)	0.11–0.08	0.16 (0.05)	0.06 to 0.26	0.23 (0.05)	0.12 to 0.35
GAD‐7	−0.01 (0.05)	−0.11 to 0.08	0.01 (0.06)	−0.12 to 0.13	−0.02 (0.07)	−0.17 to 0.12
**Step 3**						
Age	−0.01 (0.01)	−0.04 to 0.02	−0.03 (0.02)	−0.07 to 0.01	−0.04 (0.03)	−0.08 to 0.01
Sex	0.06 (0.38)	−0.69 to 0.81	0.26 (0.49)	−1.01 to 0.95	0.03 (0.57)	−1.10 to 1.17
PHQ‐9	0.10 (0.04)	0.02 to 0.18	0.06 (0.05)	−0.05 to 0.17	0.12 (0.06)	−0.002 to 0.25
GAD‐7	−0.07 (0.05)	−0.18 to 0.03	−0.05 (0.07)	−0.18 to 0.09	−0.09 (0.08)	−0.26 to 0.06
MCQ‐30 POS	NC		0.06 (0.06)	−0.06 to 0.17	NC	
MCQ‐30 NEG	0.12 (0.06)	−0.01 to 0.24	0.18 (0.08)	0.01 to 0.35	0.22 (0.10)	0.03 to 0.42
MCQ‐30 CC	NC		0.16 (0.05)	0.06 to 0.26	0.13 (0.06)	0.01 to 0.25
MCQ‐30 NC	0.08 (0.05)	−0.02 to 0.19	−0.04 (0.07)	−0.15 to 0.14	0.05 (0.08)	−0.11 to 0.22

Abbreviations: DERS‐P‐A: Difficulties in Emotion Regulation Scale‐Positive – Acceptance; DERS‐P‐G: Difficulties in Emotion Regulation Scale‐Positive—Goals; DERS‐P‐I: Difficulties in Emotion Regulation Scale‐Positive—Impulse; GAD‐7: General Anxiety Disorder‐7; MCQ‐30—CC = Metacognitions Questionnaire‐30—Cognitive Confidence; MCQ‐30—NC = Metacognitions Questionnaire‐30—Beliefs about the Need to Control Thoughts; MCQ‐30—NEG = Metacognitions Questionnaire‐30—Negative Beliefs about Thoughts concerning Uncontrollability and Danger; MCQ‐30—POS = Metacognitions Questionnaire‐30 – Positive Beliefs about Worry; NC: no significant correlations among variables; PHQ‐9: Patient Health Questionnaire‐9.

## 4 Discussion

Within the framework of the metacognitive model of emotion dysregulation (Mansueto, Jarach, et al. 2024; Wells and Matthews 1996), the aim of the present study was to extend understanding of the underlying mechanisms of positive emotion dysregulation by exploring whether metacognitive beliefs could act as potential maintenance factors of positive emotion dysregulation.

Among participants from the general population, we observed that the tendency to endorse metacognitive beliefs is associated with greater positive emotion dysregulation. Concerning the different facets of positive emotion dysregulation, our findings suggest that a higher endorsement on positive beliefs about worry, negative beliefs about thoughts concerning uncontrollability and danger, and beliefs about the need to control thoughts may be associated with a poor acceptance of positive emotions (i.e., DERS‐P Accept subscale; Weiss et al. 2015; Weiss, Darosh, et al. 2019) and with more difficulties in engaging in goal‐directed behavior (i.e., DER‐P‐ Goals subscale; Weiss et al. 2015, 2019a) and in controlling behaviors when experiencing positive emotions (DERS‐P Impulse subscale; Weiss et al. 2015; Weiss, Darosh, et al. 2019).

With regards to the clinical sample investigated, we found that a higher endorsement of negative beliefs about thoughts concerning uncontrollability and danger and lack of cognitive confidence is associated with an increase of positive emotion dysregulation. Concerning the different facets of positive emotion dysregulation, we observed that a higher endorsement of negative beliefs about thoughts concerning uncontrollability and lack of cognitive confidence were associated with greater difficulties in engaging in goal‐directed behavior (i.e., DER‐P‐ Goals subscale; Weiss et al. 2015, 2019a) and in controlling own behaviors when experiencing positive emotions (DERS‐P Impulse subscale; Weiss et al. 2015; Weiss, Darosh, et al. 2019).

Taken together, these findings mirror those of previous studies on the relationship between metacognitive beliefs and dysregulation of negative emotions (Mansueto, Jarach, et al. 2024; Mansueto, Palmieri, Sassaroli, et al. 2024; Palmieri et al. 2023), suggesting that metacognitive beliefs may play a role also in the maintenance of positive emotion dysregulation. With regard to the observed association between metacognitive beliefs and poor acceptance of positive emotions (i.e., DERS‐P Accept subscale; Weiss et al. 2015; Weiss, Darosh, et al. 2019), a possible explanation for this result may lie with previous evidence suggesting that, in response to emotional states, higher endorsement of metacognitive beliefs may lead to perseverative negative thinking about emotions (Mansueto, Jarach, et al. 2024; Spada et al. 2008), which, in turn, may lead to judge positive emotions as undesirable or frightening (Mansueto, Jarach, et al. 2024).

Concerning the observed association between metacognitive beliefs and difficulties in engaging in goal‐directed behavior when experiencing positive emotions (i.e., DER‐P‐ Goals subscale, Weiss et al. 2015; Weiss, Darosh, et al. 2019), a possible explanation for this result may lie with what has been observed in previous studies suggesting that, in response to emotional states, higher endorsement of metacognitive beliefs may reduce the ability to shift between mental sets (Kraft et al. 2017), hindering the ability to focus on the tasks at hand (Mansueto, Jarach, et al. 2024; Mansueto, Palmieri, Sassaroli, et al. 2024).

With regard to the observed association between metacognitive beliefs and difficulties in controlling behaviors when experiencing positive emotions (DERS‐P Impulse subscale; Weiss et al. 2015; Weiss, Darosh, et al. 2019), a possible explanation for this result may lie, in alignment with previous studies, with the idea that the endorsement of metacognitive beliefs, associated with activation of unhelpful control strategies (such as thought suppression and perseverative thinking), may prime the engagement in rash action and impulsive behaviors (Efrati et al. 2021; Mansueto, Palmieri, Caselli, and Spada 2024) as a means of attempting to control or reduce unwanted intrusive thoughts and the intensity of emotional states, which are a product of the engagement in unhelpful control strategies (Buyuksandalyaci Tunc and Gul 2023; Mansueto, Palmieri, Sassaroli, et al. 2024; Spada et al. 2013).

It should be noted that, among different metacognitive beliefs, in both the general population sample and the clinical sample, our findings suggest that cognitive self‐consciousness does not play a role in the maintenance of positive emotion regulation. These findings are consistent with previous studies that suggest that higher cognitive self‐consciousness may be associated with greater emotion regulation and with greater abilities to control behaviors (Diener and Wallbom 1976; Duval and Wicklund 1972; Efrati et al. 2021; Mansueto et al. 2022; Mansueto, Palmieri, Sassaroli, et al. 2024; Mansueto, Palmieri, Caselli, and Spada 2024).

Finally, comparably with previous evidence, we observed that outpatients seeking psychological treatment reported higher scores on metacognitive beliefs and positive emotion dysregulation compared with participants from the general population (Mansueto et al. 2022; Mansueto, Palmieri, Sassaroli, et al. 2024; Sun et al. 2017; Weiss, Darosh, et al. 2019; Weiss, Nelson, et al. 2019).

### 4.1 Clinical Implications

These preliminary findings bring us to consider some potential clinical implications. Firstly, in terms of assessment, information about metacognitive beliefs activated in response to positive emotional states may be gathered during the anamnesis process related with the dysregulation of positive emotions. Secondly, the metacognitive model of emotion dysregulation (Mansueto, Jarach, et al. 2024) may be used to define an idiosyncratic case conceptualization of positive emotion dysregulation and to socialize individuals to the idea that a higher endorsement of metacognitive beliefs may hinder the process of regulation of positive emotions. Participants could be socialized with the idea that, in response to positive emotions, a higher endorsement of metacognitive beliefs may be associated with poor acceptance of one's positive emotions, more difficulties in engaging in goal‐directed behavior, and in controlling own behaviors when experiencing positive emotions. Thirdly, in terms of interventions, modifying metacognitive beliefs (Wells 2011; Wells and Matthews 1996) could be considered as a potential therapeutic target for treatments aimed to reduce difficulties in the regulation of positive emotions. Metacognitive therapy techniques, such as the restructuring metacognitive beliefs, thorough metacognitive‐focused Socratic questioning, or behavioral experiments, could be suitable approaches to reduce positive emotion dysregulation (Papageorgiou 2015; Wells 2011).

### 4.2 Limitations

Results of this study must be considered with regards to its limitations. A cross‐sectional design was adopted, and this precludes drawing conclusions as to whether metacognitive beliefs play a causal role in predicting positive emotion dysregulation. Social desirability, self‐report biases, context effects, and poor recall may have contributed to errors in self‐report measurements. These limitations suggest some directions for future research. For example, an ideal demonstration of any causal contribution of metacognitive beliefs to positive emotion dysregulation could involve an experimental or clinical manipulation of metacognitive beliefs, as well as the employment of a longitudinal study designs.

## 5 Conclusion

Consistent with the metacognitive model of emotion dysregulation (Mansueto, Jarach, et al. 2024; Wells and Matthews 1996), our findings, for the first time, show that positive emotion dysregulation could be associated to the tendency to endorse metacognitive beliefs. If these findings were to be replicated, it could open‐up new prospects in the psychotherapeutic treatment of positive emotion regulation difficulties. Assessing metacognitive beliefs may allow clinicians to gain a clearer understanding of treatment trajectories to reduce difficulties in the regulation of positive emotions. Metacognitive beliefs could be the potential therapeutic target to reduce positive emotion dysregulation.

## Author Contributions

Giovanni Mansueto: designed the research; methodology; formal analysis; data curation, writing – original draft, writing – review and editing. Sara Palmieri: data curation; writing – review and editing. Lucia Salatini: acquisition of data. Sofia Piccioni: acquisition of data. Giovanni Maria Ruggiero: acquisition of data. Sandra Sassaroli: supervision. Marcantonio M. Spada: supervision; writing – review and editing. Gabriele Caselli: supervision. All authors revised the manuscript critically and provided final approval.

## Ethics Statement

The study was conducted in accordance with the Declaration of Helsinki, and the protocol was approved by the Ethics Committee of Sigmund Freud University. Informed consent was obtained from all individual subjects participating in the study.

## Conflicts of Interest

The authors declare no conflicts of interest.

## Supporting information


**Table S1.** Prevalence of psychological diseases in the clinical sample (*n* = 133)

## Data Availability

The data that support the findings of this study are available from the corresponding author upon reasonable request.
