# Supplementary material for: Positive Emotion Dysregulation: A Metacognitive Perspective
Source: Clin Psychol Psychother. 2025 Jun 26;32(4):e70109. doi: 10.1002/cpp.70109 (PMC12202843; doi:10.1002/cpp.70109)
Supplement: Supplementary file 1 — Table S1. Prevalence of psychological diseases in the clinical sample (n = 133) [file CPP-32-e70109-s001.docx]

**Table Supplementary 1.** Prevalence of psychological diseases in the clinical sample (n=133)

|  | **n (%)** |
| --- | --- |
| Major depressive disorder | 26 (19.5%) |
| Persistent depressive disorder | 13 (9.8%) |
| Bipolar Disorder | 7 (5.3%) |
| Panic disorder | 7 (5.3%) |
| Agoraphobia | 2 (1.5%) |
| Social anxiety disorder | 10 (7.5%) |
| Generalized anxiety disorder | 29 (21.8%) |
| Anxiety disorder NOS | 3 (2.3%) |
| Borderline personality disorder | 24 (18.05%) |
| Obsessive compulsive personality disorder | 8 (6.01%) |
| Narcissistic personality disorder | 6 (4.5%) |
| Avoidant personality disorder | 6 (4.5%) |
| Antisocial personality disorder | 1 (0.8%) |
| Outpatients seeking treatment for affective distress* | 24 (18.05%) |

**^*^** Outpatients with PHQ-9/GAD-7 score belove the clinical threshold.
